# The Dust Nuisance Abated

**Published:** 1860-10

**Authors:** 


### The Dust Nuisance Abated.

"Experiments," says *The London Lancet*, "have, of late, been undertaken at Lyons to allay the dust, by keeping the streets in a kind of permanent moisture. The agent used is hydrochloric acid, which prevents the rising of the dust by forming Chloride of Calcium, a deliquscent salt."

Query?—Would not water be better and cheaper where the surface of streets are composed of granite, clay and sand?

